# The 10-year evolution of a dental undergraduate ‘Blended Learning’ suturing programme

**DOI:** 10.1038/s41405-026-00467-4

**Published:** 2026-09-12

**Authors:** Anna Dargue, Richa Arya, Tamara Khayatt

**Affiliations:** 1 Department of Oral Surgery, Bristol Dental School, Bristol, UK; 2 Department of Oral Surgery, Bristol NHS Foundation Trust, Bristol, UK

**Keywords:** Dental education, Oral surgery

## Abstract

**Introduction:**

Undergraduate dental students in the United Kingdom must be competent in intra-oral suturing before graduating. In 2014, a blended learning programme was introduced to enhance suturing teaching.

**Methods:**

Students first completed an online suturing tutorial. This was designed for them to review at their own pace, promoted self-directed learning and provided a degree of standardisation in theoretical knowledge. Subsequently, students attended a practical session using simulation aids, translating theory into practice and promoting deeper learning. Staff offered guidance in these sessions and students undertook peer evaluation. Mixed methods action research was used iteratively to study student evaluation from the programme over a decade.

**Results:**

745 undergraduate student responses were evaluated. These influenced adaptations to the programme such as introducing teaching earlier in the undergraduate programme, mounting suturing foam pads into a phantom head, and extending the duration of the session. 2024 results demonstrated 98% of students felt competent to handle the suture needle, and 95% felt prepared to apply these skills on a patient after completing the programme.

**Conclusions:**

Student evaluation over a decade has driven continuous development of the programme. Our study demonstrates incremental changes have enriched the learning experience and preparedness for clinical practice.

## Introduction

The ability to place a functional suture to control bleeding [1] or repair soft tissue following a dental extraction is an essential skill every student needs to master prior to qualification. Suturing contributes to several of the General Dental Council’s (GDC) learning outcomes, as well as the competencies outlined for a graduating European dentist [2,3]. Suturing is one of the most challenging skills to acquire, and proficiency requires practice and repetition [4,5]. UK dental experts have noted that a potential consequence of poor clinician confidence in suturing could be increased referrals, so dental schools have a responsibility to prepare students for clinical practice [6].

In undergraduate medical education, a systematic review of suture teaching noted a great diversity in terms of timing, instructor level and nature of delivery, and that these factors all influenced the quality of learning. All studies had a didactic component followed by practical teaching [4]. A wide variety of methods to teach psychomotor skills are available, including photos, models, videos, live demonstrations and haptic virtual technology [6,7]. Demonstrations in dental education are seen to be crucial to enable visualisation and better understanding of procedures [7]. Teaching suturing on bench models rather than on patients saves money, time, faculty resources and addresses ethical and patient safety concerns [8]. The minimum instructor: student ratio should be 1:4, allowing time for staff to provide personalised feedback, an important variable for supporting technique development [8]. Regular reinforcement over time is recommended and improves skills retention [9].

A 2011 study found that in 7 UK dental schools, students were mostly taught suture skills in years 3 or 4 with a combination of seminar or tutorial, followed by practical teaching using suture pads [10]. Teaching took place over 1-5 hours with a staff: student ratio of between 1:4 and 1:6 [10]. Studies have found that most UK dental students have satisfactory suturing skills [10,11].

Until 2014, undergraduates at Bristol Dental School were taught suturing skills in year three of a five-year programme, as part of a broader surgical skills practical session using pigs’ jaws. Staff: student ratio was 1:8 - 1:10, with 60 to 80 students in each year. The theory of suturing was covered later in a year four lecture on basic surgical principles.

In 2014, a novel suturing programme was developed. It aimed to improve quality, consistency and efficiency of teaching suturing skills, and minimise disruption to clinical sessions by utilising an evidence-based, ‘flipped classroom’ type of blended learning [12–15]. The programme consisted of an online tutorial followed by face-to-face, small group, practical skills teaching of suturing techniques using desktop models and foam pads mounted in a phantom head. Student feedback was acquired via a questionnaire, promoting reflective educational practice and facilitating the action research process [16,17]. The questionnaire collected both quantitative and qualitative data as part of mixed methods research [18].

**Fig. 1:**
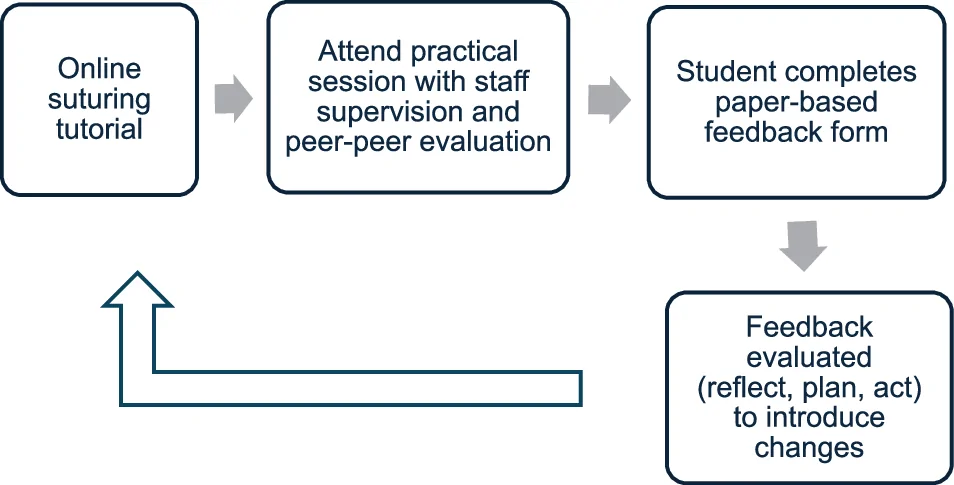
Summary diagram to show action research process for annual course development.

**Fig. 2:**
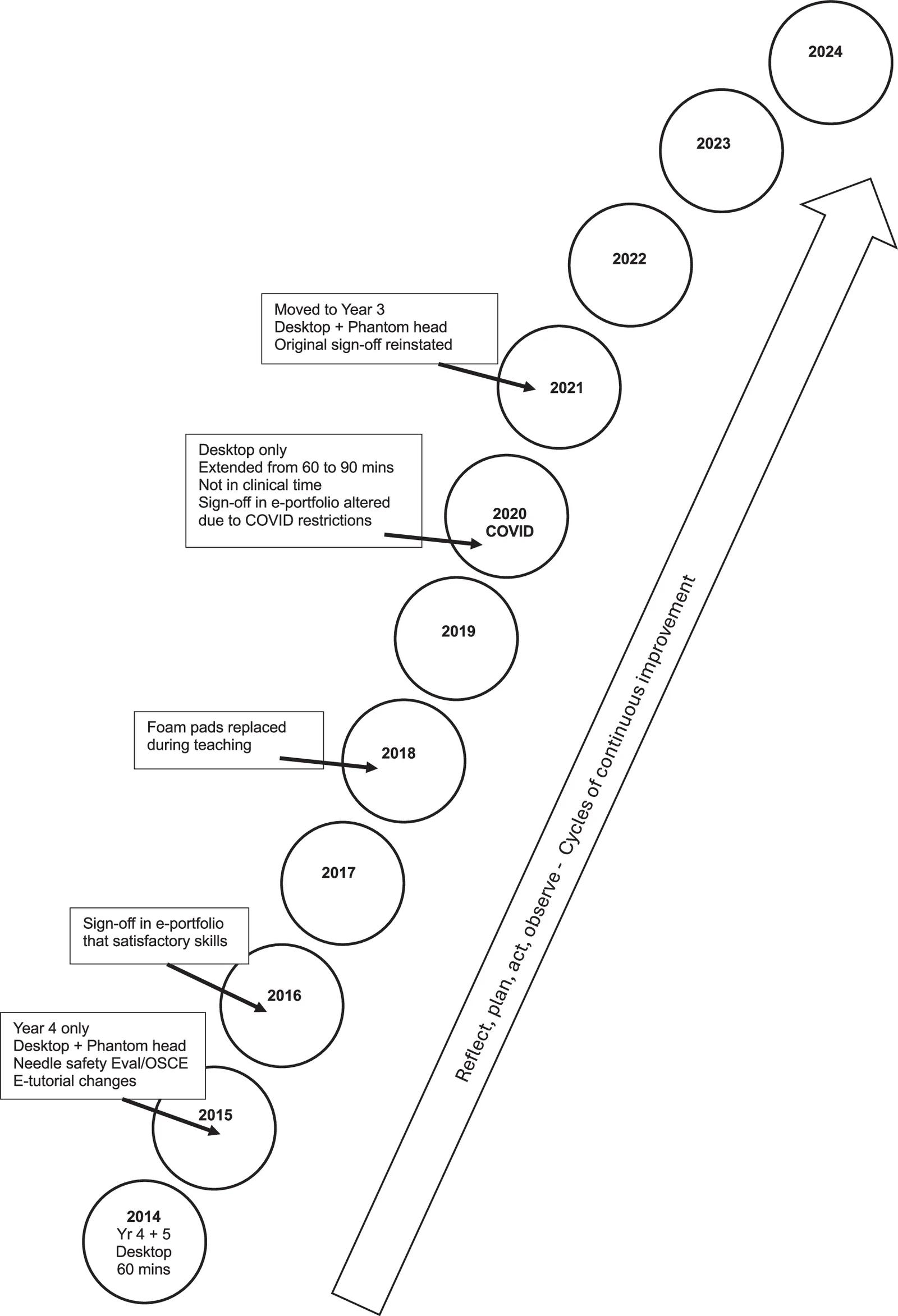
Diagram to show timeline of changes made to the suturing programme.

Action research is defined as the continuous interaction between the research process, with action, reflection and evaluation [19]. In this study, it involved student evaluation of the teaching activity, staff reflection on student evaluation, followed by action to implement changes to improve the suturing programme, then further reflection on the effectiveness of this change (see Fig. 1). Fig. 2 presents a summary of the timeline and the evolution of changes made over the decade to improve teaching based on student feedback.

This study outlines how mixed methods action research was employed from the programme’s introduction in 2014 and continued over a ten-year period to influence programme development.

## Methods

This was a retrospective study involving a review of educational activity with students knowingly assisting in improvements to the teaching course [18]. The University of Bristol research ethics committee deemed that this was an educational evaluation activity and under Health Research Authority guidance, ethical approval was not required [20]. As a voluntary, anonymous survey of dental professionals, completion of the survey implied consent to participate and participants were able to withdraw at any point while completing the survey. The evaluation data collected was obtained without coercion from students and contained non-sensitive questions.

An online tutorial covering the importance of competence in, theory of and techniques used in suturing was created. Consistent with the “flipped classroom” model, this allowed students to review content at their own pace and promoted self-directed learning. Its primary use is as preparation for the practical session, but it can also be used during the practical session as a guide or as a retrospective revision aid. Self-assessment questions, with individual feedback to promote active learning through interaction, were also included.

Students were prompted via email to complete the online tutorial a week prior to the practical.

**Fig. 3:**
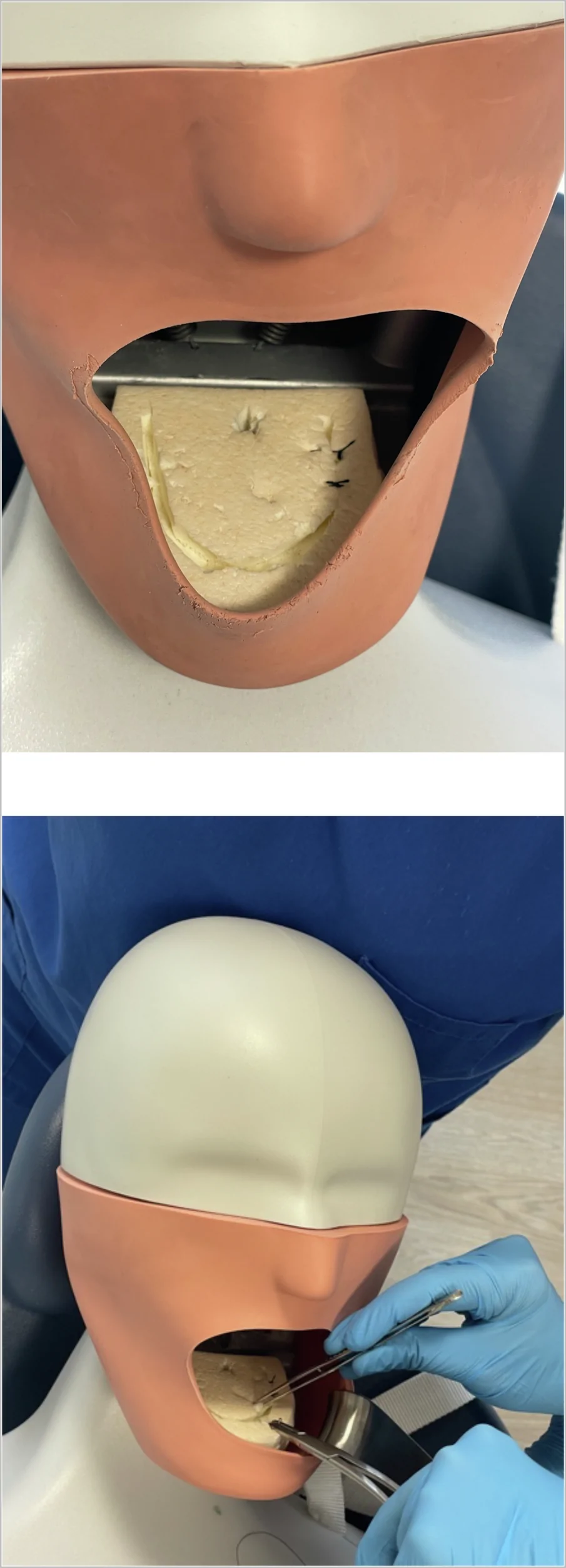
Images of foam pads fitted into phantom head and in use during suture practice.

A one-hour practical session was subsequently delivered with a 1:4 or 1:5 staff: student ratio. Peyton’s four-step method for teaching skills was used as the basis for placement of an interrupted suture using desktop skin pad models [21]. Peyton’s teaching approach is a stepwise approach for procedural skills following the four steps of: demonstration by the teacher, deconstruction into smaller steps, comprehension as the learner explains the steps and finally the learner performance [21]. Following student feedback requesting more realistic suturing settings, staff developed an additional, cost-effective simulation using a phantom head. Skin pads (limbsandthings.com) [22] were prepared to a dental arch template with sections of the foam cut away to resemble extraction sockets (see Fig. 3). The modified suture pads were screwed onto the Frasaco [23] phantom head. Students then practised with close staff supervision and support, and real-time feedback. Peer-to-peer evaluation was also encouraged using a “12 salient steps” proforma (see Table 1) to allow each student to observe and assess their peer’s suturing technique. The peer evaluation proforma used was identical to the mark scheme used by staff in the summative Objective Structured Clinical Exam (OSCE) mark scheme and provided constructive alignment [24].

**Table 1:** Twelve salient steps proforma used by students for peer evaluation

1. Needle located in holder half to two-thirds of the way along the length.
2. Tissue everted using Gillies tissue forceps.
3. Needle enters tissue perpendicular to wound edge.
4. Use of hand pronation and supination when passing needle through tissue.
5. Same size bite on each side of wound.
6. 2 throws clockwise across needle holders.
7. 1 throw anticlockwise across needle holders.
8. Each throw is down square, either by having the sutures crossed, or by crossing the hands (i.e. the suture is laid flat).
9. Appropriate tension of suture across wound
10. Knot positioned on one side of wound.
11. Suture ends cut to length of approximately 5 mm.
12. Needle safety throughout placement and knotting.

All students were invited to complete an anonymous, paper-based evaluation questionnaire at the end of the practical session (see Table 2). The six quantitative statements used a 5-point Likert scale (1 as strongly disagree to 5 as strongly agree) response. An additional statement was added in 2015. Free responses were allowed for the two qualitative questions (see Table 2.).

**Table 2:** Student questionnaire for evaluation of the suturing programme

Quantitative questions:
1. I completed the online tutorial before attending today’s practical session. 2. I feel the online tutorial helped to support today’s practical session. 3. Today’s practical session built on and advanced my learning after the online tutorial. 4. I feel that evaluating another student’s suturing helped my understanding of suturing. 5. The staff gave me helpful feedback. 6. I feel competent to handle and dispose of the suture needle safely. (Question added in 2015.). 7. I feel prepared to see my first patient for suturing.
**Qualitative questions:** 1. Which parts of the online tutorial and practical session have been most helpful for your learning?. 2. Do you have any comments on what we could improve, and how do you suggest we accomplish this?

This mixed methods study used a convergent design where both qualitative and quantitative data were collected in parallel and analysed at the same time. Simple analysis of percentages and trends was performed on quantitative data, and qualitative data was analysed using thematic analysis [25]. The first author was responsible for initial coding and grouping into themes. Themes were then reviewed and discussed at teaching meetings on an annual basis. In line with the iterative process of action research, staff reflected and implemented changes to improve the programme based on the agreed themes (see Fig. 1) [19]. A pragmatic philosophical approach underlines this mixed-method action research with a need to design and implement effective action plans to improve teaching [26].

## Results

A total of 745 undergraduate student evaluation responses were received between 2014 and 2024, with an average annual response rate of 89% (range 69 to 100%)

### Quantitative findings

**Fig. 4:**
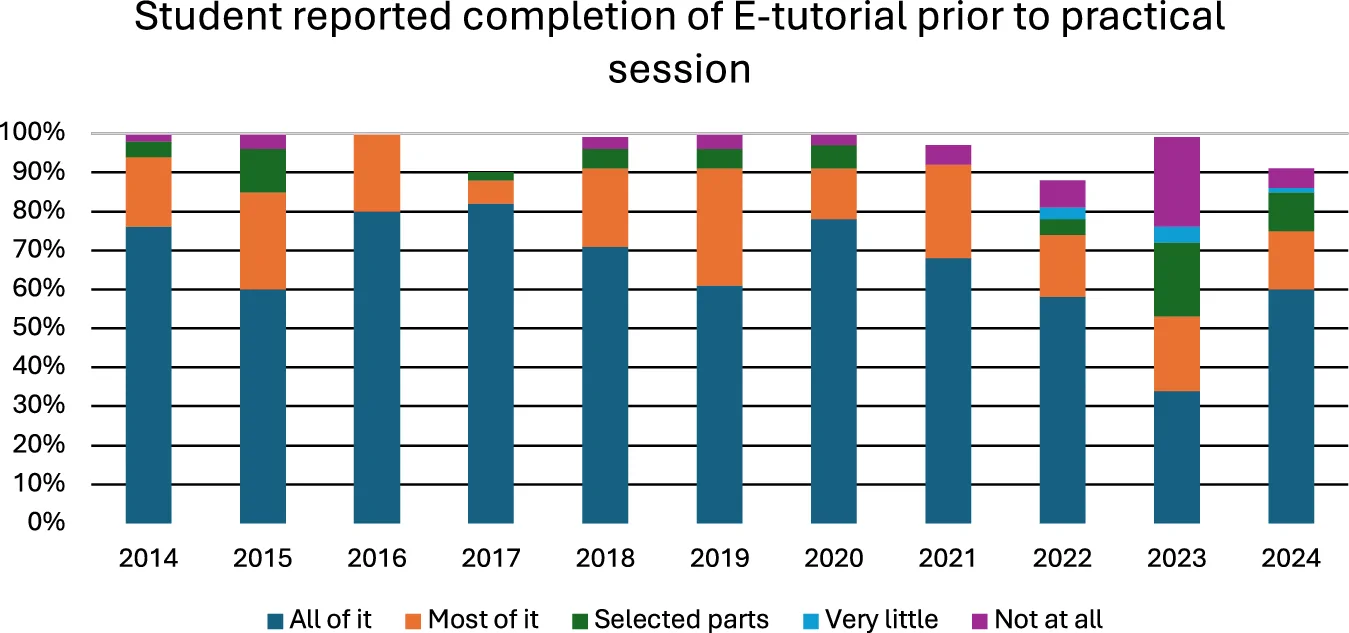
Bar chart to show student-reported completion of the online tutorial content prior to the practical session.

**Fig. 5:**
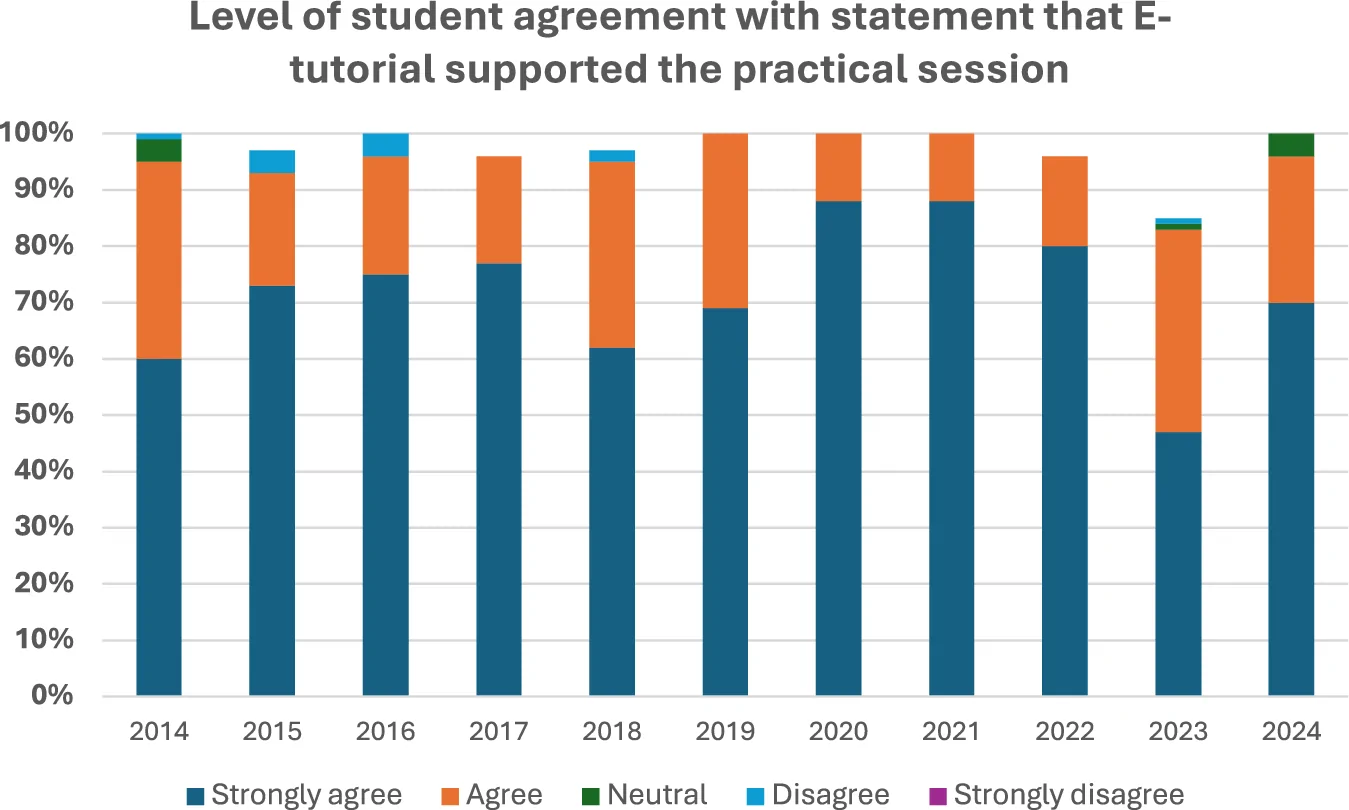
Bar chart to demonstrate the level of student agreement with the statement that the practical session advanced their learning after the online tutorial.

**Fig. 6:**
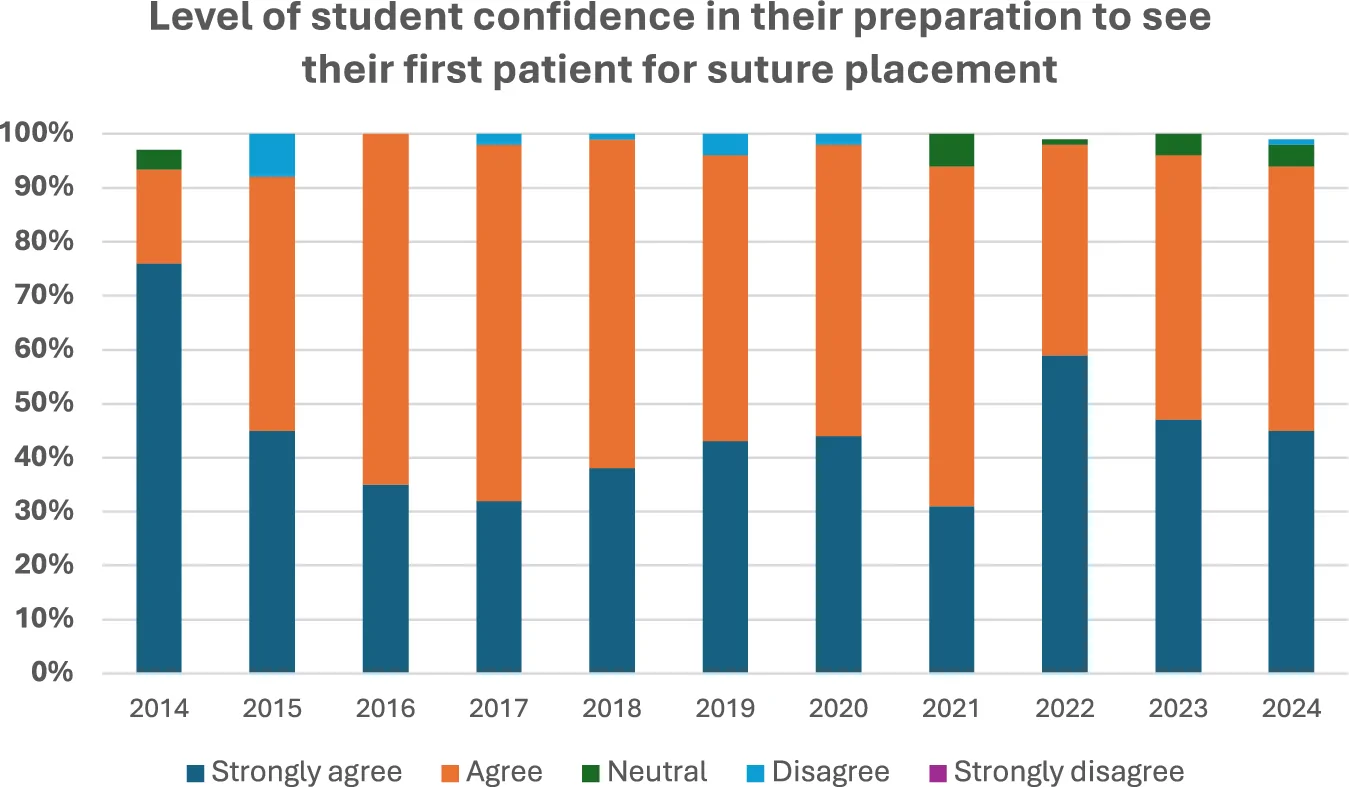
Bar chart to show students’ level of confidence in their preparation to see their first patient for suture placement.

Over the decade, two-thirds of students completed all modules of the online tutorial before attending the practical session. Fig. 4 shows the amount of the online tutorial that students stated they reviewed prior to the practical session. A particularly low completion rate for the online tutorial was seen in 2023 (34% completed the full online tutorial) and was accompanied by a poorer student opinion that the practical was supported by the online tutorial (47% strongly agree). Just over two-thirds of students strongly agreed and 23% agreed that the online tutorial helped to support the practical session over ten years. Similarly, 79% of students strongly agreed and 16% agreed that the practical session helped to advance their learning after the online tutorial. Fig. 5 demonstrates the students’ view of the practical session over the decade. Students strongly agreeing that the practical session advanced their learning increased by 30% between 2014 and 2024, which may reflect how the modifications made enhanced student learning. On average, around half of students strongly agreed, 33% agreed and 12% felt neutral when asked whether evaluating a peer's suturing helped to develop their understanding of suturing. Staff were consistently considered to provide helpful feedback, with an overall average of 97% of students strongly agreeing/agreeing to this statement. Since 2015, all students on average either strongly agreed (83%) or agreed (17%) that they felt competent to safely handle and dispose of the suture needle. In terms of preparedness to see their first patient, an average of 44% of students strongly agreed, 50% agreed and just 3% disagreed that they felt prepared. Fig. 6 demonstrates student’s level of confidence in their preparation for clinical suture placement over ten years of data collection. In 2015, 8% of students disagreed vs 1% in 2024, which may suggest that the gradual changes made to the programme helped to increase student confidence in feeling prepared.

### Qualitative findings

Thematic analysis of open-ended responses revealed five key themes.

### Usefulness of videos in online tutorial

Students praised clear, detailed video demonstrations and appreciated the ability to revisit the content flexibly.

*“Videos are very useful to see actually how to do it and good to refer back to.” (2014)*

The speed, structure and types of videos that had been created helped demystify the skill.

*“Slowed down videos and voice-overs in the online tutorial were really useful and helpful to visualise.” (2024)*

### Blended learning advantages

The combination of online preparation with hands-on practice was repeatedly cited as advantageous.

*“Online tutorial was excellent preparation. The practical strongly reinforced the theory in the online tutorial.” (2020)*

### Simulation model realism

While the suture pads were helpful initially, students preferred the positive change made to the course where students had to suture within the phantom head. This change was made following student suggestions for improvement and made suturing more realistic.

*“Practising on a phantom head after we had learnt on the suturing sponge was really useful – helped get used to holding and positioning.”* (2023)

### Value of peer and staff feedback

Students noted the positive impact of staff feedback on their skill development and learning, as well as peer-evaluation encouraging self-reflection.

“*Staff feedback and support throughout the session.”* (2015)

“*Working in pairs – learning from your partner.”* (2014)

### Timing and duration

Several students requested earlier delivery of teaching from 4th year to 3rd year, so they could apply their skills to patients.

*“Do this before pigs heads (4th* *year) as I now feel more confident.”* (2017)

Students also requested longer sessions to allow more practice and build confidence.

*“More than an hour practice would be better!”* (2018)

## Discussion

Overall, student evaluation shows a very positive view of the suturing programme. The average response rate of 89% is notably good over a ten-year period, with 745 students voicing their opinions out of a total population of 840, so results are deemed representative. The percentage change across each of the seven statements was a gradual improvement year-on-year (see Figs. 4–6). Staff considered variation within and between student cohorts and whether changes made an impact on the suturing programme.

Over ten years, 83% of students had reviewed all or some of the online tutorial prior to attending the practical session. Other dental undergraduate studies have found lower completion rates of online material prior to flipped classrooms, with less than 50% reviewing all the material [27]. Almost all students (95% agreed/strongly agreed) found the online tutorial helped support the practical session. The advantages for students in online learning have been detailed by many experts: consistency in information, pace, timing, and location encourage self-direction and ownership of learning, as well as flexibility to access content again to reinforce knowledge [4,7,13–15,28–32]. There are also benefits for educators surrounding sustainability and accountability of online learning being easy to update and distribute [29,31] Motivation is stated to be one of the struggles for online learning as students need to self-motivate to engage with the content and especially for blended learning, students need to review the online tutorial to benefit from the subsequent in-person session [32]. Thus, experts recommend careful planning with thoughtful design to improve engagement [32]. Interactive quizzes, videos, and a clear structure were included to ensure active, learner-centred experiences. The introductory section explained why mastering suturing skills was important to trigger the emotional component of learning, which is known to have a powerful effect [33]. The student evaluation reflects the considered design of the online tutorial with positive reviews on quizzes, a user-friendly structure and content engagement.

Videos were particularly highly rated by our students as being beneficial for their learning. Evidence in the literature highlights videos produce significant improvement to both practical skills and knowledge compared to live demonstrations, due to enhanced visualisation with greater detail and the flexibility for student review as needed [7]. A study offering a choice of different videos for medical students to learn to suture, found that the slow speed video with expert narration, was the most consumed [34]. This was also confirmed by our students; slowed down videos were particularly valuable for learning along with the possibility of revisiting the videos.

The blended learning approach was very positively received, with an average of 95% of students agreeing/strongly agreeing over the decade that the practical session advanced their learning following the online tutorial. There are multiple individual studies that demonstrate the successful use of a blended learning format within undergraduate dental education [12,28–30,35]. A meta-analysis examining the evidence for flipped classrooms in healthcare education (including dentistry) found a statistically significant improvement in learning performance compared with traditional methods [13]. Specifically for dental education, a systematic review of the effectiveness of flipped learning, found it was at least as effective to conventional learning [15]. Dental students also enjoy this type of teaching format, finding it fun, interactive and collaborative [12,14,15]. The comments made by our students concur with this, validating this learner-centred approach. It is our assertion that thoughtful integration of a blended learning programme takes advantage of the benefits of online learning, followed by the more efficient use of tutors’ teaching time. This is particularly relevant in a period of austerity with decreasing funds, increasing student numbers and difficulty recruiting and retaining staff [30,36].

Following the introduction of the programme, useful student suggestions were made requesting more ‘realistic’ suturing opportunities which resulted in changes to the course (Fig. 2). Foam suture pads were shaped into ridges and secured within the phantom heads to better simulate the challenges of intra-oral suturing (Fig. 3). This facilitated translational learning of knowledge into practice and student evaluation highlighted the advantage of this more realistic simulation. A systematic review on teaching suturing skills to medical students found low fidelity, ‘dry’ desktop models were just as effective as high fidelity, ‘wet’ models [4]. However, given the technical challenges of suturing within the oral cavity, we argue that dental students need to be more skilled at suturing than their medical student colleagues. This review’s findings should therefore be considered in this context. Our low-cost solution aimed to better replicate the limited space and shape of the mouth. Phantom heads offer several benefits for simulation skill training over the clinical setting, including standardisation, resource control, and most importantly, patient safety [37]. It also allows students the opportunity for confidence-boosting repetitive practice [37].

On annual review of student evaluation in 2015, staff noted 8% of students disagreed with the statement that they felt prepared to suture clinically (see Fig. 6). Staff also felt more focus was needed around needle safety after delivering the course for the first time. Consequently, in 2016, a formal, e-portfolio competence sign-off was introduced. Staff confirmation of ability appeared to build confidence in the undergraduates and additional teaching was offered to students when needed. Needle safety was also added to both the peer evaluation and OSCE marking scheme, as well as adding a question to the student evaluation form. Education encouraging the adoption of safe habits early on is important in minimising the risk of sharps injury [38]. Over the decade, all our students felt competent (100% agreed/strongly agreed) to handle and safely dispose of the suture needle. The course appears to be achieving its aim of preparing students to safely suture in patients’ mouths.

Peer evaluation was generally appreciated by students; however, the quantitative data demonstrates more variability in its impact on learning. The structured proforma issued aimed to support feedback. Student evaluation particularly highlighted the importance of staff feedback in increasing confidence and providing encouragement. An increase to over 90% of students strongly agreeing that staff gave helpful feedback was noted from 2020 onwards. This coincided with an increase in the duration of teaching, highlighting a positive change to the programme. Experts emphasise that appropriate personalised feedback from staff is the most important variable for facilitating motor learning, especially in the initial stages of suturing skill development [8].

The iterative increase in session length from sixty to ninety minutes and earlier introduction of suturing teaching in the third rather than fourth year of the five-year programme, reflect the responsiveness of staff to student needs following suggestions for improvement (Fig. 2.) These changes also supported more opportunities for practising suture skills during clinical sessions and using the pigs head surgical skills teaching in the fourth year to reinforce learning. There is strong evidence that distributed practice (spaced repetition) is a more effective method of learning and retaining skills, when compared to massed practice [4,9].

This study has several limitations. Students may possess biases, both explicit and implicit, which influence their responses when completing course evaluation forms. Students hold socially constructed assumptions of what constitutes effective teaching and if their expectations remain unfulfilled, they can respond with critical feedback [39]. Classroom paper-based evaluations have a favourable setting bias on results when compared to internet-based evaluation, and therefore the results should be interpreted with caution [40]. Students were given time in class to complete the questionnaire, which encouraged better response rates, and is reflected in the high 89% average response rate. This increases the validity of the results. It is also recommended to look at overall trends and patterns, as occurred in this study, when interpreting evaluation rather than individual results. Researcher bias is possible due to a single researcher undertaking thematic analysis on the qualitative data with a risk of subjectivity. To mitigate this risk, student evaluation data was shared with the teaching team (10 individuals) for data triangulation, which increases credibility, and potential changes to the course were discussed. Data were from a large cohort in one dental school with a good response rate and a sustained improvement in the results over the years. The findings are likely to have external validity and be transferable to teaching suturing skills to dental students in the UK. Ensuring rigour in action research can be difficult due to its practical nature. Nonetheless, this study took place over a prolonged period, and there was the possibility of reviewing changes made to the course and their impact over several years, making the study more robust.

Future research into the teaching of suturing skills to dental undergraduates could consider the use of peer teachers, as this has been used effectively in medicine [4,6]. Further areas of interest could include using 3D printed models or haptic virtual technology to enable more life-like suture settings [7,41].

## Conclusions

This suturing programme was developed using a blended learning format based on proven and considered educational principles. Our study demonstrates how the iterative process of using student evaluation through mixed methods action research can positively shape a teaching course over a decade. The combination of both qualitative and quantitative data offered a more complete understanding of students’ views about the suturing programme. Our findings appear to demonstrate that the modifications made to the course were successful, with most students feeling prepared for clinical practice and fewer suggestions for improvement over the decade. Whilst the findings have limited generalisability to other courses, the concepts seen highlight possibilities for other educational settings in terms of advancing course review and the importance of meticulous course design.

## Data Availability

Data cannot be shared openly but are available upon reasonable request from the authors.
